# Microbiota Changes Due to Grape Seed Extract Diet Improved Intestinal Homeostasis and Decreased Fatness in Parental Broiler Hens

**DOI:** 10.3390/microorganisms8081141

**Published:** 2020-07-28

**Authors:** Jeremy Grandhaye, Veronique Douard, Ana Rodriguez-Mateos, Yifan Xu, Alex Cheok, Antonella Riva, Rodrigo Guabiraba, Olivier Zemb, Catherine Philippe, Magali Monnoye, Christophe Staub, Eric Venturi, Alix Barbe, Christelle Ramé, Joelle Dupont, Pascal Froment

**Affiliations:** 1 INRAE UMR85 Physiologie de la Reproduction et des Comportements, 37380 Nouzilly, France; jeremy.grandhaye@inrae.fr (J.G.); alix.barbe@inrae.fr (A.B.); christelle.rame@inrae.fr (C.R.); joelle.dupont@inrae.fr (J.D.); 2 Micalis Institute, INRAE, AgroParisTech, Université Paris-Saclay, 78350 Jouy-en-Josas, France; veronique.douard@inrae.fr (V.D.); catherine.philippe@inrae.fr (C.P.); magali.monnoye@inrae.fr (M.M.); 3 Department of Nutritional Sciences, School of Life Course Sciences, Faculty of Life Sciences and Medicine, King’s College London, London SE1 7EH, UK; ana.rodriguez-mateos@kcl.ac.uk (A.R.-M.); yifan.yu@kcl.ac.uk (Y.X.); alex.cheok@kcl.ac.uk (A.C.); 4 INDENA, 38 Avenue Gustave Eiffel, 37095 Tours, France; antonella.riva@indena.com; 5 Infection and Innate Immunity in Monogastric Livestock, Centre INRAE Val de Loire, ISP UMR1282, 37380 Nouzilly, France; rodrigo.guabiraba-brito@inrae.fr; 6 UMR 1388 GenPhySE, Université de Toulouse, INRAE, INPT, ENVT, 31320 Castanet Tolosan, France; olivier.zemb@inrae.fr; 7 INRAE Unité Expérimentale de Physiologie Animale de l’Orfrasière UEPAO 1297, 37380 Nouzilly, France; christophe.staub@inrae.fr (C.S.); eric.venturi@inrae.fr (E.V.)

**Keywords:** broiler, grape seed, microbiota, adipose tissue

## Abstract

In poultry, the selection of broilers for growth performance has induced a deterioration in the health of the parental hens associated with poor reproductive efficiency. To improve these parameters, we administered to laying parental broiler hens a regular diet supplemented or not (Control) with a moderate (1%) or a high level (2%) of grape seed extract (GSE). The 1% GSE diet was administered from a young age (from 4 to 40 weeks of age) and the high level of 2% GSE was administered only during a 2-week period (from 38 to 40 weeks of age) in the laying period. The analysis of 40-week-old hens showed that 2% GSE displayed a reduction in the fat tissue and an improvement in fertility with heavier and more resistant eggs. Seven monomer phenolic metabolites of GSE were significantly measured in the plasma of the 2% GSE hens. GSE supplementation increased the relative abundance of the following bacteria populations: *Bifidobacteriaceae, Lactobacilliaceae* and *Lachnospiraceae*. In conclusion, a supplementation period of only 2 weeks with 2% GSE is sufficient to improve the metabolic and laying parameters of breeder hens through a modification in the microbiota.

## 1. Introduction

Since the 1960s, commercial broilers have been selected for growth performance [1]. However, the parent stock of commercial broiler chickens have displayed a rapid alteration of their health status associated with elevated inflammation and fattening, joint problems and alteration of cardiac function and welfare. The consequences on broiler breeder hens are more pronounced than in males, since the hens are kept alive beyond 40 weeks. One of the features of the change in hen health status is the development of excess adiposity, which induces chronic inflammation and oxidative stress associated with the impairment of numerous cellular and tissue functions [2]. Moreover, genetic selection and ad libitum feeding cause excess of energy [3], leading to an aberrant follicular recruitment, dysregulation on pre-ovulatory follicle selection, inducing multiple ovulation, leading to poor reproductive efficiency [4,5]. To limit the alteration of metabolic and reproductive parameters during the laying period, the diet composition is adjusted to prevent excess adiposity [6] and to ameliorate laying rate and eggshell strength [7,8,9,10,11,12].

A change in dietary composition is frequently achieved through supplementation with antioxidants, among which selenium, vitamins and (poly)phenols are the most frequently used. Phenolic compounds are found in plant products (fruits, vegetables, flowers) and when metabolized by the organism they have been reported to have multiple biological effects in addition to their antioxidant properties [13,14]. Grape products such as grape juice, grape pomace or grape seeds are good sources of flavonoids and phenolic compounds; grape seed extracts (GSE) are of particular interest because they are easily usable in livestock diets. These compounds have potential health benefits such as reducing bone resorption or cardiovascular risk factors [15,16,17]. Recently, microbiota have been shown to play a role in the bioavailability of grape products, and gut bacteria may play a key role in mediating or potentiating their health effects [18,19,20]. Among the flavonoids present in GSE, the small intestine readily absorbs monomers such as catechins, but oligomers or polymers are not absorbed and reach the colon where they are metabolized by gut bacteria into phenolic acids and therefore absorbed by the colonic cells [21,22]. Some studies have reported the beneficial effects of grape product supplementation in broilers. Hence, chicks supplemented for 35 days with grape (grape extract, grape pomace, or GSE) showed reduced mortality rates and lesion scores after *E. tenella* infection [23]. Dietary supplementation with grape seed oil (21 days) also improved the quality of broiler chicken meat [24]. However, a high concentration of GSE supplementation (5 g/kg for 21 days) can have deleterious effects by reducing feed conversion in broilers and consequently stunting growth [25]. However, to our knowledge, no data have focused on the effect of a GSE supplemented diet on female parental broiler hens during the reproductive period.

GSE can also modify the microbiota composition [26]. (Poly)phenols may change intestinal microbial composition by selectively inhibiting the growth of some pathogenic bacteria while enhancing the growth of probiotic bacteria [27]. In mice, a diet enriched in high-flavonoid apples increased the number of total bacteria by 6% [28] and the supplementation of a high fat diet with proanthocyanidin specifically decreased the relative abundance of *Lactococcus* and *Bacteroides* [29]. Several studies showed a positive link between gut microbiota modification in response to GSE and improvement of specific metabolic parameters such as adiposity and inflammation [29]. The role of the gut microbiota in regulating body composition and metabolic outcome in obese mice and humans has been well established [30,31]. However, little is known about the role of microbiota in growth performance and body composition in broilers. In hens, the modification of microbiota could also influence reproductive performance. Indeed, the use of a probiotic mixture of *Bacillus subtilis* and *Enterococcus faecium* increased the egg production rate, the egg weight, eggshell strength, and albumen height in laying hens [32].

The objective of the present study was to investigate in parental female broiler the effect of a GSE supplemented diet on performance and metabolic parameters linked to fatness. The caecal microbiota composition and its metabolic adaptation to the GSE was also analyzed in order to decipher its potential role in mediating the GSE effects. In order to optimize future dietary supplementation procedures, two different approaches were used: a long period of dietary supplementation during almost the entire life of the hens with a low amount of GSE and a short period of GSE exposure (2 weeks during the laying period).

## 2. Materials and Methods

### 2.1. Animals

Thirty-six female parent broiler breeders (Cobb 500) from a commercial breeding unit of Hendrix Genetics (Saint Laurent de la Plaine, France) were studied until they were 40 weeks old. All experiments were approved by the Ethics Committee in Animal Experimentation of Val de Loire CEEA Vdl registered by the National Committee ‘Comité National de Réflexion Ethique sur l’Expérimentation Animale’ under the number 19 (APAFIS# 10237-201706151202940v3). All experiments were performed in accordance with the European Communities Council Directive 2010/63/UE.

The 36 parental hens were distributed in 3 homogeneous groups of 12 hens. Each group was divided into 2 pens of 3 m^2^ (6 hens/pen). The animals were reared in rooms initially bedded with 8 cm of clean shavings or hulls at the Experimental Unit of the Poultry Experimentation Center of Tours (UEPEAT, Nouzilly, France) according to the conventional conditions of breeding. Light conditions were 14 h of light per day (minimum light intensity of 20 lux) at their arrival followed by a gradual decrease to approximately 8 h at the 1st week, kept constant until the age of photostimulation (21st week) and then a gradual increase until reaching 14 h of light per day at the end of study (40th week) every 4 weeks. Housing temperature began at 31 °C and was decreased gradually to 21.5 °C, with a range of humidity between 35 and 70% during the 40 weeks. Ten hens per group were selected to measure the bodyweight, and the thickness of back fat by ultrasound.

The fat composition of the chickens was estimated by dorsal ultrasound (MyLab 30 Gold Vet ultrasound scanner, Hospimedi France, Saint-Crépin-Ibouvillers, France) equipped with 2 linear probes (Esaote L332 and L435) as described in [6]. Only the measurements at the 40th week of age are represented. At the 38th and 39th week of age, all eggs were collected and individually weighed (KERN Scale—ref. PCB 250–3, Kern and Sohn, Balingen, Germany). The analysis of laying rate (number of egg per hen per day) and egg weight were performed every day in all pens (36 hens in total). Eggs were recovered 2 times per day for 2 weeks and each egg was weighed; in total, at least 120 eggs per group were measured. Each day, the broken eggs were noted and were reported to daily egg production per group.

At 40 weeks, all hens were stunned by electrocution and killed by exsanguination as recommended by the ethical committee. The blood samples were collected in heparin tubes, centrifugated to keep the plasma (1500 g, 10 min, 4 °C), aliquoted and stored at −20 °C until analysis. The cecal digesta were weighted and stored at −80 °C until analysis. The cecum tissue (100 mg) was washed in the phosphate buffered saline (PBS), then stored at −80 °C.

### 2.2. Nutrition and Feeding Strategies

From the first day to the 4th week, all birds received an ad libitum diet (free access to food). From the 4th week to the 40th week of age, chicks received three different diets according to Hendrix Genetics’ recommendation: grower (from week 4 to 18), prebreeder (from week 18 to 21) and breeder diets (from week 21 to 40). At the 4th week, two groups of hens were separated in function of 2 types of diets: control diet and the same diet supplemented with GSE at 1% of total diet. The GSE supplement was provided by INDENA (Tours, France) The composition of GSE was analyzed using high performance liquid chromatography (HPLC) by INDENA, and this showed that the most important component was the procyanidins (> 90%). At week 38 of age, a third group was created from the control group and received a diet supplemented with 2% of GSE for two weeks. In order to adjust the amount of feed consumed by animals, animals in the control pens were weighed and then feed was adjusted weekly with respect to the theoretical curve provided by the supplier. The resulting three groups were: control, n = 12; long time 1% GSE supplemented diet from week 4 to 40 of age, n = 12; and a short time 2% GSE supplemented diet from week 38 to 40 of age, n = 12 (protocol in Supplemental Data 1).

### 2.3. Analysis of Plasma (Poly)Phenols by Ultra Performance Liquid Chromatography Coupled with Triple Quadrupole Mass Spectrometry (UPLC-Q-q-Q MS)

The chicken plasma samples were first hydrolyzed with β-glucuronidase/sulfatase from Helix pomatia (Type 1H, Sigma-Aldrich, Saint Louis, MO, USA). The identification and quantification of (poly)phenol metabolites in plasma after GSE consumption was performed using micro-elution solid phase extraction (μ-SPE), as previously described [33,34], followed by UPLC-Q-q-Q MS. The detection of target metabolites was performed on a SHIMADZU Triple Quadrupole Mass Spectrometer (LCMS-8060, SHIMADZU, Kyoto, Japan) through an electro-spray ionization (ESI) source in negative mode. Extracted samples (5 μL) were injected through a Raptor Biphenyl column (2.1 × 50 mm, 1.8 µm, Restek, Bellefonte, PA, USA) with a compatible Raptor Biphenyl Guard Cartridge (5 × 2.1 mm, Restek, Bellefonte, PA, USA) in the UPLC system following a previously validated separation method [30]. Authentic standards were injected for the identification and quantification of individual (poly)phenols. Recoveries of the compounds during μ-SPE were estimated with the isotopically labeled internal standard (±) catechin-2,3,4-^13^C3 (Sigma-Aldrich, Steinheim, Germany). Analysis of the plasma (poly)phenols were performed on 4 hens from (control) group, 5 hens from (1% LT) group and 5 hens from (2% ST) group.

### 2.4. Microbiota DNA Extraction and 16S RNA Sequencing

DNA from bacteria was extracted from caecum content (12 samples per group) using G’NOME DNA isolation kit (MP Biomedicals, Strasbourg, France) [35]. V3–V4 region of the 16S rRNA genes was amplified using MolTaq (Molzym, Plaisir, France), 50 ng DNA and the primers V3F: TACGGRAGGCAGCAG and V4R: ATCTTACCAGGGTATCTAATCCT [36]. Purified amplicons were sequenced using the MiSeq sequencing technology (Illumina) at the GeT-PLaGe platform (Toulouse, France). Paired-end reads obtained from MiSeq sequencing were analyzed using the Galaxy-supported pipeline named FROGS (Find, Rapidly, OTUs, Operational Taxonomic Units) with Galaxy Solution) [37]. For the preprocessing, reads with length ≥ 380 bp were kept. The clustering and chimera removal tools followed the guidelines of FROGS [37]. Assignation was performed using SILVA132 16S pintail100. OTUs with abundances lower than 0.005% were removed from the analysis [38].

### 2.5. Tissue RNA Extraction and Reverse Transcription Reaction

Total RNA from caecal tissue was extracted by using the TRIzol^®^ reagent (Invitrogen, by Life Technologies, Villebon sur Yvette, France). Total RNA (1 µg) was reverse transcribed with 0.5 mM of dNTP, 2 M of RT-Buffer, 0.5 μg/µL of OligodT, 0.125 U of ribonuclease inhibitor and 0.05U of RT-MMLV (Promega, France) for 1 h at 37 °C (Thermocycler PE9700, Perkin Elmer, France). cDNA was stored at −80 °C until qPCR. Biological samples from 10 hens per group were analyzed

### 2.6. Real Time PCR

Real-time PCR was performed using the MyiQ Cycle Device (Bio-Rad, Marnes-la-Coquette, France), in a mixture with 9 µL SYBR Green Supermix 1X Reagent (Bio-Rad, Marnes-la-Coquette, France), 0.23 µL of specific primers (250 nM) as indicated in (Supplemental Data 2), 5.45 μL of cDNA diluted 1:5 in water, and 5 µl H2O for a total volume of 20 μL. The samples were set up in duplicate on the same plate according to the following procedure: after an incubation of 2 min at 50 °C and a denaturation step of 10 min at 95 °C, samples were subjected to 40 PCR cycles (30 s at 95 °C, 30 s at 60 °C, 30 s at 72 °C), followed by the acquisition of the melting curve. Primers’ efficiency (E) was performed from serial dilutions of a pool of obtained cDNA and ranged from 1.8 to 2. as described in [12]. Three reference genes (GAPDH, EEF1A1 and *ACTB*) were used. For each gene, expression was calculated according to primer efficiency and quantification cycle (Cq), where expression = E^−Cq^. The relative expression of the gene of interest was analyzed to the relative expression of the geometric mean of the three reference genes.

### 2.7. Protein Extraction and Western Blotting

Total protein was extracted from the caecum (100 mg) in lysis buffer and exposed to 3 repeated freeze/thaw cycles. The protein concentration in the supernatants were determined using a colorimetric assay kit (DC assay kit; Uptima Interchim, Montluçon, France). The proteins extracted (80 μg) were denatured, subjected to SDS-PAGE in a 12% polyacrylamide gel, transferred onto nitrocellulose membranes and incubated at 4 °C overnight with the following antibodies: P53 and IL6 produced by Agro-Bio (Agro-Bio, La Ferté Saint Aubin, France). The targeting peptides are EFIQETFDSEKQNVESLC from IL6 (gallus gallus, NCBI Reference Sequence: NP_989959.1) and peptide CEGNPQARYHDDETTKRK from P53 (gallus gallus, NCBI Reference Sequence: NP_990595.1). All antibodies were used at 1:1000 dilution in Western blotting. The signal was detected by enhanced chemiluminescence (Amersham Pharmacia Biotech, Orsay France) and the signals were quantified by using ImageJ software (NIH, Bethesda, MD, USA). The results are expressed as the intensity signal in arbitrary units, after normalization by an internal standard (tubulin, Sigma-Aldrich Saint-Louis, MO, USA). Analysis was performed on biological samples from 5 hens per group

### 2.8. Oxidative Stress Analysis

ROS-Glo™ H_2_O_2_ Assay (Promega, Charbonnieres, France) was performed on plasma to analyze oxidative stress (10 biological samples/group). Assays were realized according to the manufacturer’s instructions. Briefly, samples were stressed with H_2_O_2_ Substrate Solution for 3 h, then, samples were incubated 20 min with ROS-Glo™ Detection Solution in the dark to stabilize the luminescent signal. The plate was measured using a microplate reader, Luminoskan Ascent (VWR International, France) to record luminescence.

### 2.9. Metabolites

Phospholipides, cholesterol, triglycerides, uric acid, calcium, and glucose were measured by using the following spectrophotometric assays, respectively (Biolabo, Maizy, France), and lactate concentration by a Sigma-Aldrich kit (Sigma-Aldrich Saint-Louis, MO, USA). ATP concentrations and caspase 3 activity were measured by using the CellTiter-Glo™ ATP Assay Kit and Caspase-Glo™ Assay (Promega, Charbonnieres, France), respectively, according to the manufacturer’s instructions. Measurement were performed on biological samples from 10 hens per group.

### 2.10. Hormones

Concentration of adiponectin and chemerin in plasma were measured by ELISA (MyBioSource ADPN ELISA kit: Chicken Total Adiponectin ELISA Kit, MyBioSource Chicken CML ELISA Kit, San Diego, CA, USA) [39]. Measurement were performed on biological samples from 10 hens per group.

### 2.11. Statistical Analysis

One-way ANOVA test using GraphPad Prism 8 (La Jolla, CA, USA) was used to evaluate differences between groups. The results are expressed as mean ± SEM. Values were determined to be significant when * *p* < 0.05, ** *p* < 0.01, *** *p* < 0.001 or by different letters indicating significant difference between groups (*p* < 0.05).

16S sequencing data were analyzed using the Phyloseq [40], ggplot2 [41] R packages in additions to custom scripts. Samples were rarefied to even sampling depths before computing within-samples compositional diversities (observed richness and Inverse Simpson) and between-samples compositional diversity (Bray–Curtis and Jaccard dissimilarities). Principal coordinate analysis (PCoA) was performed on Bray–Curtis and Jaccard dissimilarities. Raw, unrarefied OTU counts were used to produce relative abundance graphs. Observed richness and inverse Alpha diversity data were analyzed using 1-way ANOVA. A permutational multivariate analysis of variance (PERMANOVA) test was performed on the Bray–Curtis and Jaccard matrices using 9999 random permutations and at a significance level of 0.01. Phylum and family relative abundances were compared using a Kruskal–Wallis test followed by Dunn’s test.

## 3. Results

### 3.1. GSE Diet Influences Metabolism

**Figure 1:**
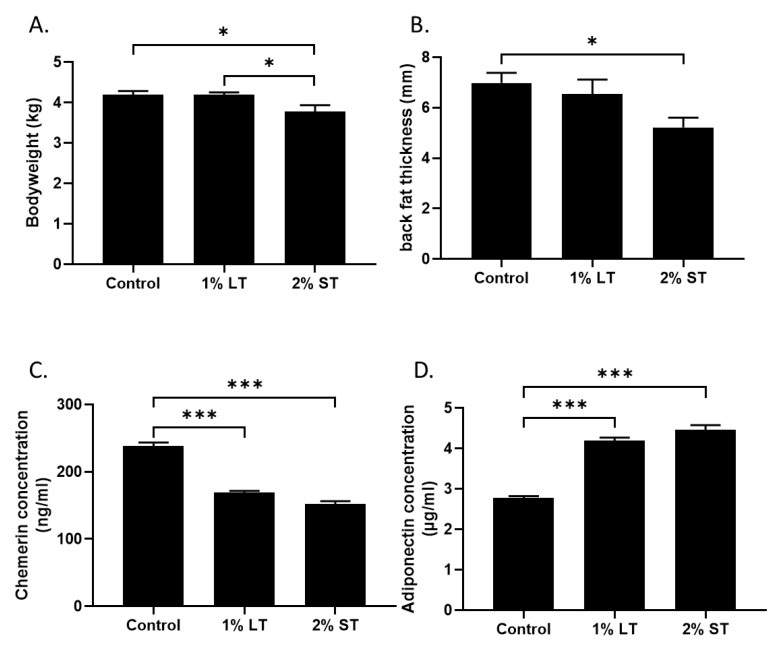
(**A**) Bodyweight of 3 groups (Control; 1% LT; 2% ST) at 40th week of age. (**B**) The thickness of back fat (mm), as a marker of fat mass, was measured by ultrasonography. (**C**,**D**) Plasma concentrations of chemerin and adiponectin were measured at 40th week to evaluate the metabolic status of the animals. (n = 10 animals/group). Control, 1% Long Treatment, 1% LT; 2% Short Treatment, 2% ST. *, *p* < 0.05; ***, *p* < 0.001, significant differences with the control.

First, the addition of 2% GSE for 2 weeks (short treatment, 2% ST, from 38 to 40 weeks of age) in the diet decreased significantly (*p* < 0.05) the body weight of hens (Figure 1A) as well as the fatness measured by ultrasound compared to the control group (Figure 1B). The group that received 1% GSE (long treatment, 1% LT, from 4 to 40 weeks of age) did not show changes in body weight or fatness compared to control (*p* > 0.05). In order to clarify the mechanism underlying the decrease in adiposity and body weight in the hens of the 2% ST group, we focused on two hormones produced by adipose tissue. The adiponectin plasmatic levels were lower and chemerin plasmatic levels were higher in both GSE groups (Figure 1C,D, *p* < 0.001) when compared to the control group. Then, the blood lipid profile was assessed (Table 1). The phospholipid levels decreased significantly (*p* < 0.05) in the plasma of both GSE groups when compared to the control group, while cholesterol and triglycerides levels remained unchanged (*p* > 0.05). The uric acid concentration was significantly (*p* < 0.01) reduced in both GSE groups when compared to the control group. Calcium concentration was significantly (p < 0.01) lower in the hens of the 2% GSE group when compared to the two other groups. After 8 h of starvation, lactate and glucose levels were similar in all groups. Importantly, the blood reactive oxygen species (ROS) levels were lower in hens of the 2% GSE group than in those of the control group, confirming the antioxidant properties of GSE (*p* < 0.05).

### 3.2. Plasma GSE Phenolic Metabolites

To determine the metabolic outcome of dietary GSE, we quantified the plasmatic levels of 13 phenolic metabolites (Table 2). The levels of (+)-catechin (*p* < 0.01), m-coumaric acid (*p* < 0.05), p-coumaric acid (*p* < 0.01), (-)-epicatechin (*p* < 0.01), 3-hydrobenzoic acid (*p* < 0.01), (-)-epicatechin-3-O-methylether (*p* < 0.001) and 3-hydroxyphenylacetic (*p* < 0.01) increased significantly in the plasma of the hens fed 2% ST when compared to the control group. The levels of protocatechuic acid, syringic acid, 2-hydroxybenzoic acid, 2,4-dihydroxybenzoic acid, 4-hydroxybenzoic acid and (-)-epicatechin-4-O-methylether did not differ among the three groups (*p* > 0.05). Interestingly, the long-term treatment with a lower amount of GSE did not lead to any increase in the level of any of the phenolic metabolites.

### 3.3. Caecal Microbiota Composition

**Figure 2:**
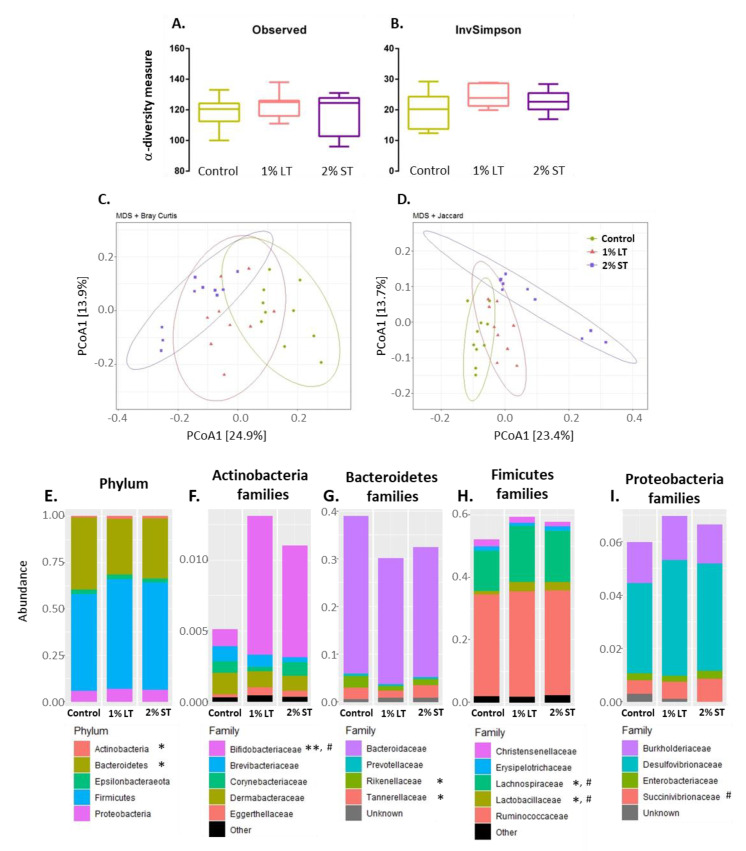
Microbiota composition of the caecum content collected at 40th week of age from hens of 3 groups (Control; 1% LT; 2% ST). Evolution of -diversity represented by observed species richness (**A**) and Inverse Simpson Index (**B**). Principal coordinates analysis (PCoA) multi-dimensional scaling (MDS) of Bray–Curtis (**C**) and Jaccard (**D**) compositional dissimilarity between samples. Average relative abundance at phylum (**E**) and family (**F**–**I**) level in the caecal content of each group. Observed species richness and Inverse Simpson Index values are means ± SEM compared by one-way ANOVA followed by Tukey’s post hoc test. Phylum and family relative abundance data were compared using the Kruskal–Wallis test; * indicates significantly different abundances between Control and 1% LT (with * *p* < 0.05, ** *p* < 0.01), # between Control and 2% ST (with # *p* < 0.05). Control, 1% Long Treatment, 1% LT; 2% Short Treatment, 2% ST.

In order to characterize the caecal microbiota in response to the GSE-enriched diet, we measured diversity indices and determined the caecum microbiota composition at the phylum and family taxonomic levels. At the operational taxonomic unit (OTU) level, the richness did not change among the three groups of hens (Figure 2A), nor did the Inverse Simpson Index, indicating no change in the number of effective taxa in response to GSE enrichment (Figure 2B). The β-diversity analysis based on Bray–Curtis and Jaccard dissimilarity revealed changes in the caecal microbial community in the presence of GSE in the diet (*p* < 0.05) (Figure 2C,D). At the phylum level, the relative abundance of *Proteobacteria*, *Firmicutes* and *Epsilonbacteraeota* was not significantly affected by GSE enrichment, while the relative abundance of *Actinobacteria* significantly increased in the 1% LT group of hens at the expense of the *Bacteroidetes* (Figure 2E). Analysis at family taxonomical levels revealed that this enhancement in the phylum of *Actinobacteria* in response to GSE diets was entirely supported by the increase in the relative abundance of *Bifidobacteriaceae*, which increased significantly in both the 1% LT and 2% ST groups (Figure 2F). In the phylum of the *Bacteroidetes*, the 1% LT treatment significantly decreased the relative abundance of two minor families, *Rikenellaceae* and *Tannerellaceae* (Figure 2G). Within the *Firmicutes*, both GSE-enriched diets induced a modest but significant increase in the relative abundance of the *Lactobacilliaceae* and *Lachnospiraceae* families (Figure 2H). In the phylum of the *Proteobacteria*, the relative abundance of *Succinivibrionaceae* increased modestly in the caecal content of the hens of the 2%ST group (Figure 2I).

### 3.4. Analysis of Inflammation Markers in Caecal Tissue

**Figure 3:**
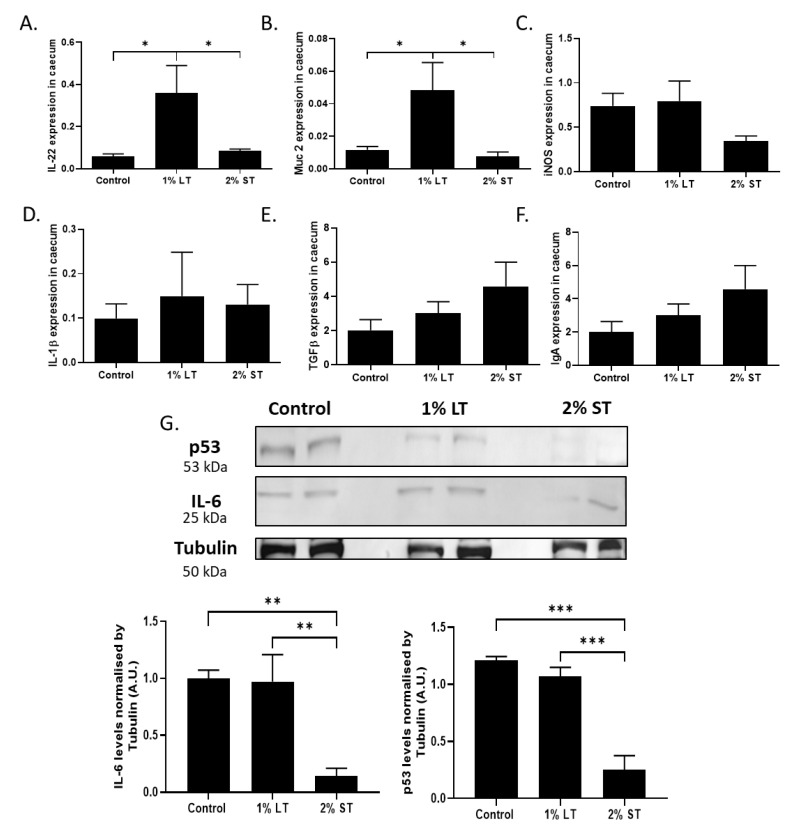
Anti-inflammatory gene expression (**A**) IL-22, (**B**) Muc 2, (**C**) iNOS in the caecal tissue collected at 40^th^ week of age from hens (group: Control; 1% LT; 2% ST) as assessed by qPCR. Inflammatory gene expression (**D**) IL-1β, (**E**) TGFβ, (**F**) IgA in the caecal tissue collected at 40th week of age from hens (group: Control; 1% LT; 2% ST) as assessed by qPCR. (n = 10 animals/group) (**G**) Effect of the grape seed extract (GSE) on the protein level of p53 and IL6 in the caecal tissue as quantified by Western blot. Equal protein loading was checked by reporting the membrane with an anti-tubulin. Representative blots from caecal tissue of the 3 groups are shown, (n = 5 animals/group). Control, 1% Long Treatment, 1% LT; 2% Short Treatment, 2% ST. *, *p* < 0.05; **, *p* < 0.01; ***, *p* < 0.001, significant differences with the control.

The expression of genes related to gut homeostasis and inflammation was studied in the caeca to evaluate inflammation of intestinal mucosa in the animals. Expression of genes coding for interleukin 22 (IL-22), mucin 2 (Muc 2), tumor growth factor beta (TGF-β) and immunoglobulin A (IgA), which are related to mucosal anti-inflammatory regulation, and inducible nitric oxide synthase (iNOS), interleukin 1 beta (IL-1β) and interleukin 6 (IL-6), genes linked to pro-inflammatory responses, were analyzed. Expression of IL-22 and Muc 2 increased significantly (*p* < 0.05) in the caecal epithelium of the hens fed 1% GSE from 4 to 40 weeks of age (Figure 3A,B). Despite a tendency for a lower iNos expression level in the caecum of the hens fed 2% GSE for 2 weeks, no significant difference was found among the three groups (Figure 3C). For the expression of inflammation gene: interleukin 1 beta (IL-1β), tumor growth factor beta (TGF-β) and immunoglobulin A (IgA) (Figure 3D–F), no difference was observed between the groups. However, a lower level of IL-6 was measured in the caecal epithelium of the hens from the 2% ST group when compared to the control group (Figure 3G, IL-6: *p* < 0.01).

### 3.5. Laying Parameters

**Figure 4:**
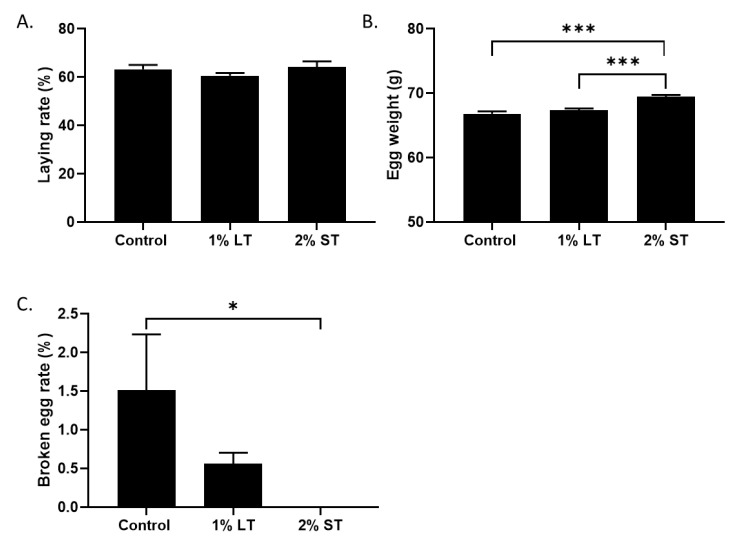
Percentage of laying rate during the collection period (**A**). Egg weight (g) (**B**) and solidity (**C**), collected daily for 2 weeks (group: Control; 1% LT; 2% ST). *, *p* < 0.05; ***, *p* < 0.001; significant differences with the control. (n = 12 animals/group with at least 120 eggs per group).

The eggs were collected during the last two weeks of both treatments. The supplementation of the diet with GSE did not modify the laying rate of the hens (Figure 4A), but, in the 2% ST group, the eggs were heavier (Figure 4B, *p* < 0.001) compared to control and 1% LT group, and displayed an increase in shelf strength compared to those of the control group (Figure 4C, *p* < 0.05)

## 4. Discussion

**Figure 5:**
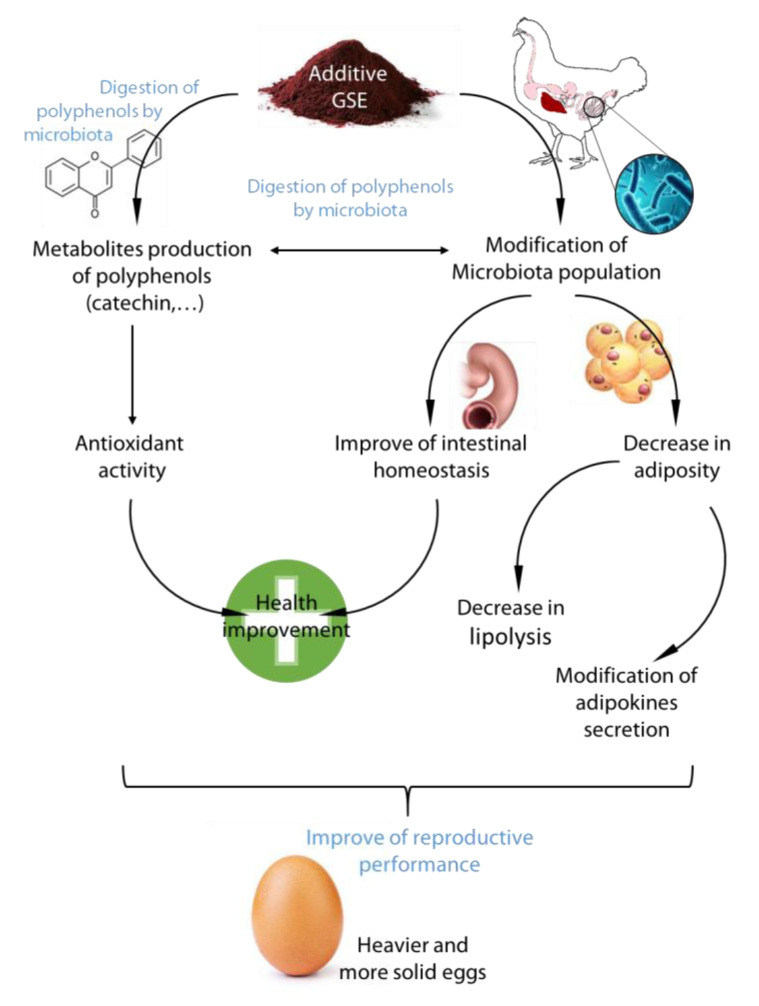
Schema representative of the effects of GSE on microbiota population and adipose tissue in reproductive hens. Representative figure of the diet supplementation of GSE on microbiota population, intestinal homeostasis, and adiposity in reproductive hens. GSE modifies the bacteria content, but bacteria are involved to digest poly and oligomers of polyphenols present in GSE into active monomers with antioxidant activity. The change in the microbial/(poly)phenols environment decreased lipolysis and adipose tissue and improved intestine homeostasis and the antioxidant status.

The objective of the present study was to evaluate the effects of a long or short exposure to GSE on physiological parameters of parental broiler hens during the reproductive period. The growth physiological and metabolic parameters evaluated demonstrated that GSE addition in the food improved antioxidant status, decreased the fattening of animals and modified the bacterial population in the caecum in association with an improvement of some parameters of intestinal homeostasis (Figure 5).

### 4.1. Time/Concentration Exposure

Previous studies on the effect of grapes on broilers have aimed to focus on meat quality or growth performance. A supplementation of 2.5 g of GSE/kg to 21-day-old chicks had no adverse impact, while 5 g/kg retarded their growth rate and decreased their feed conversion [25]. However, the use of grape pomace (5, 10, and 20 g/kg) in the diet of broilers from hatching to 40 days old increased growth and feed conversion [42]. These studies showed that the way in which grapes are incorporated into the diet (GSE or grape marc) can have different effects on animal growth. Furthermore, in these cases, supplementation of the grapes was carried out for short periods and not during the breeding period.

In our study, two doses of 10 g/kg or 20 g/kg of GSE were used during the growth and all adulthood or for a short period of time (2 weeks) only during the reproductive period. Small physiological impacts were observed with 1% GSE supplementation for 40 weeks, while the use of 2% GSE for only 2 weeks induced strong changes, specifically by decreasing the fatness, which may be associated with the previous study cited before, showing that a high dose of GSE was associated with growth retardation [25]. The lower fatness was associated with a slight decrease in bodyweight as well as reduced phospholipid and adipokine level. In a previous study, we showed that the measure of back fat thickness by ultrasound is a good predictive marker of the adipose tissue content in the whole body of broilers [6]. Furthermore, in another study, we showed a negative correlation between adiponectin concentrations and fattening, but also a positive correlation with chemerin concentrations in plasma and fatness [43]. So, the use of GSE in the diet could decrease adiposity in female broilers during the reproductive period. Reduction in the oxidative stress and fatness could improve the longevity and the quality of the reproductive period of the parental broiler hens.

### 4.2. Microbiota and GSE

The effects on growth in response to an average dose are all the more surprising, since most of the (poly)phenols contained in the grape seed are highly polymerized proanthocyanidins that are difficult to assimilate [44]. They could be absorbed only as monomers (catechin, epicatechin) or oligomers (proanthocyanidin) [45]. The monomers are produced by the degradation of GSE by gut microbiota. In rats, grape polyphenols (procyanidin B1 or procyanidin A2) are converted into dimers by the intestinal microorganisms [46]. In pigs, 6 days of feeding with 1% GSE is associated with an increase in secondary monomer metabolites, such as (-)epicatechin, (+)-catechins or 3-hydroxybenzoic acid, in the feces [47]. In chickens, 2.5% to 5% of a whole grape extract supplementation was also associated with an increase in microbial-derived phenolic metabolites ((+)-catechin or (−)-epicatechin) in ileal digesta and excreta [48].

In the present study, we measured phenolic compounds in the blood, thus showing whether or not GSE is digested by the microbiota. Interestingly, 2% ST GSE supplementation led to elevated levels of by-products of polyphenol, likely resulting from proanthocyanidin degradation by gut microbiota like catechins or epicatechins, whereas 1% LT GSE supplementation had no effect. The use of 1% GSE in the diet may have been insufficient to be detected significantly in the plasma. Even the control group displayed low levels of phenolic compounds, likely originating from the wheat and soya of the diet [49]. These increases in by-products of polyphenols in animals with 2% GSE were associated with a better antioxidant status, as observed by the low ROS content in plasma. The antioxidant characteristics of grape, grape products and their by-products contain scavenging free radicals which form some complexes with metal ions to prevent and reduce the development of ROS [50]. Catechins have been described as possessing great antioxidant potential and can decrease oxidative stress in the organism by its ROS scavenger activity or by its potential role in the induction of antioxidant enzymes or the inhibition of pro-oxidant enzymes [51]. The ROS level is a reflection of the stress of an animal [52,53]. Several studies have shown that a decrease in the oxidative status of an animal can be associated with a decrease in fattening [54,55]. The analysis of the body composition revealed a decrease in the bodyweight [56]. However, several markers associated with the diminution of the adipose tissue mass have been shown after 1% or 2% GSE exposure: reduction in the dorsal fattening level by ultrasound [6], weight of adipose mass, modification in adipokine levels: chemerin decrease and adiponectin increase [43], decrease in phospholipids and uric acid levels in plasma. Uric acid level has also been shown to be a marker of adiposity, because high secretion was observed in obese mice [57]. Similar observations have been measured in caeca tissue with low phospholipids, triglycerides, cholesterol and ATP, suggesting a decrease in lipogenesis (Supplemental Data 3). Two hypotheses for the decrease in fattening could be suggested: firstly, a direct effect of GSE (and its by-products) on different cells (adipose tissue, muscle, etc.); or secondly, a change in microbiota population [58]. Indeed, some findings have shown that obesity is correlated with the *Firmicutes/Bacteroidetes* ratio [29,59,60]. In these studies, the researchers showed that the increase in *Firmicutes* and the decrease in *Bacteroidetes* were associated with obesity. However, in the present study, the level of *Firmicutes* did not vary and a decrease in *Bacteroidetes* was observed. Although the abundance of *Firmicutes* is similar, the composition of bacteria in this phylum was modified with an increase in *Lactobacillus* levels. *Lactobacillus* used as a probiotic in the diet of mice or rats can attenuate the overweight and elevate the adiponectin secretion associated to the excess of adipose tissue [61,62]. Studies have shown that grape and its by-products (like catechin) can stimulate the growth of *Lactobacillus* in the intestine of animals [63,64]. Thus, the modification of the microbiota by GSE could be a cause of the reduction in the fat storage in hens [65]. Finally, the increase in by-products of polyphenols in plasma, caused by the consumption of 2% GSE during a short period, can be associated with a decrease in adiposity and an improvement in welfare.

In addition to its ability to enhance the bioavailability of (poly)phenols, the gut microbiota composition adaptation to the GSE supplementation could also contribute to the improvement of physiological and metabolic parameters. First of all, the changes observed in the composition of the microbiota were more pronounced in the group that received 1% GSE for 36 weeks than in the group that received 2% GSE for 2 weeks.

The results show that the *Actinobacteria* population increased and *Bacteroidetes* decreased in hens exposed to 1% GSE for 36 weeks. *Bacteroidetes* have been associated with negative health outcomes such as irritable bowel syndrome, diarrhea and chronic inflammatory bowel disease [66,67]; therefore, the decrease in *Bacteroidetes* in the caeca of hens improves the intestinal health in our study. GSE supplementation (1 or 2%) was also associated with the increase in the relative abundance of *Lactobacillus*, *Lachnospirales* and *Bifidobacteriaceae*. These data are in agreement with earlier studies that characterized the intestinal microbiota adaptation to GSE in livestock animals [47,68]. *Lactobacillus* is used as a probiotic in broilers, since it has been shown to have a beneficial effect on growth, carcass quality and to prevent intestinal infection [69]. *Lactobacillus reuteri* KUB-AC5 exposure immediately after hatching allowed long-term enrichment in *Lactobacillus* and long-term reduction in *Proteobacteria* and other non-beneficial bacteria [70]. As previously described, *Bifidobacteria* also boost carbohydrate digestion [71] and improve intestinal heath by reducing diarrhea or inflammatory bowel disease in humans [72,73,74,75]. These data obtained with the use of GSE in the diet were part of an improvement in welfare.

### 4.3. Improvement of Caecal Homeostasis

The modification in the bacterial population and presence of polyphenols in the caecum appeared to improve the intestinal homeostasis with a better antioxidant status and decrease in the expression of pro-inflammatory factors. The results are associated with a higher expression of IL-22 and Muc-2 for the long GSE exposure. Indeed, assimilable phenolic compounds such as catechin are able to regulate the growth of some bacterial species, probably via an interaction with enterocytes activity [76]. The short exposure at the 38th week of age is possibly too late (at adult age) to measure an effect on gene expression by epithelial or goblet cells. However, this hypothesis needs to be confirmed in the future.

In mice, IL-22 is known to ameliorate intestinal homeostasis by enhancing STAT3 activation and the expression of mucus-associated molecules by goblet cells to limit intestinal inflammation [77]. The mucus barrier plays such a fundamental role in intestinal functions and homeostasis; Muc-2 also promotes the production of mucin and has an active role in regulating mucosal immunity [78]. The use of GSE at 0.1% GSE (w/v) for 12 weeks in female mice increased the colonic goblet cell density that was associated with increased mRNA expression of Muc-2 [79].

The pro-inflammatory markers, IL-1β and IgA, play a central role in the intestinal inflammation amplification cascade by increasing the permeability of the intestinal epithelial junction [80]. We noted that these genes are not affected by GSE exposure, whatever the time of exposure, but IL6, identified as a crucial regulator of inflammatory responses [81], diminished significantly with 2% GSE exposure for 2 weeks. The markers of cell death measured by p53 [82] or cleaved caspase 3 activity were at lower levels in the presence of GSE, reinforcing the well-being status of intestine cells and good homeostasis.

## 5. Conclusions

Nowadays, the use of natural products in food is of great interest for the development of agroecology strategies for breeders and to conserve broiler performances. However, studies on broilers have been performed on up to 35-day old chickens but not on adults in the reproductive period. In our study, grape and, more particularly, grape seed extract were studied in the diet [56,83]. The antioxidant property of GSE has been reported to improve the total antioxidant status in the plasma of Japanese quail [84] and antioxidant capacity in the blood of broilers [85].

In this study, we have shown that a diet enriched in GSE during the reproductive period can be beneficial for intestine health, which impacts different functions of the organism (behavior, health, reproduction) (Figure 5). In brief, until changes in the selection of broiler lines appear, a short-term choice could be the use of GSE in the diet to improve animal welfare (adiposity and oxidative status). The long-term choice could be to improve the inflammatory status of the gut. Moreover, this study opens up new directions by selecting specific probiotics based on the modifications of the microbiota observed, which could mimic the beneficial effects of GSE in hens. Finally, this strategy of using natural molecules in the diet of a highly selected line (broiler) over a long breeding period for this breed (40 weeks) has shown beneficial effects quickly, and could be of use for other strains of poultry, bred for muscle quality or egg production.

## Figures and Tables

**Table 1:** Analysis of the plasma concentrations at 40th week of age of phospholipids, cholesterol and triglycerides, lactate, glucose, uric acid, calcium and reactive oxygen species (ROS) levels, ROS in the 3 groups (control, 1% LT, 2% ST); 1% Long Treatment, 1% LT; 2% Short Treatment, 2% ST. (n = 10 animals/group).

	Control	1% LT	2% ST
Phospholipides (g/L)	1.91 ± 0.04 ^a^	1.74 ± 0.02 ^b^	1.75 ± 0.05 ^b^
Cholesterol (g/L)	1.80 ± 0.06 ^a^	1.92 ± 0.12 ^a^	1.83 ± 0.08 ^a^
Tryglycerides (g/L)	7.09 ± 0.28 ^a^	7.03 ± 0.28 ^a^	6.71 ± 0.29 ^a^
Lactate (µg/mL)	109.86 ± 33.81 ^a^	177.5 ± 25.58 ^a^	106.68 ± 29.59 ^a^
Glucose (mg/dL)	194.84 ± 28.26 ^a^	186.36 ± 32.47^a^	189.86 ± 39.80 ^a^
Uric acid (mg/L)	96.57 ± 4.08 ^a^	79.55 ± 3.82 ^b^	76.60 ± 2.21 ^b^
Calcium (mg/L)	135.71 ± 4.11 ^a^	151.39 ± 4.62 ^a^	106.47 ± 7.05 ^b^
ROS levels (relative luminescent units)	0.83 ± 0.04 ^a^	0.75 ± 0.07 ^ab^	0.62 ± 0.05 ^b^

a,b in superscripts indicate significant differences between group (*p* < 0.05).

**Table 2:** Plasma concentrations (ng/mL) at 40th week of age of the following (poly)phenol metabolites: (+)-catechin, protocatechuic acid, p-coumaric acid, syringic acid, (-)-epicatechin, 2-hydroxybenzoic acid, 5-hydroxybenzoic acid, m-coumaric acid, 2,4 dihydroxybenzoic acid, 4-hydroxybenzoic acid, (-)-epicatechin-3-O-methylether, (-)-epicatechin-4-O-methlether, 3-hydrocyphenylacetic acid. (n = 4 animals for control and 5 animals in 1% Long Treatment, LT and 2% Short Treatment, ST groups.

Values (ng /mL)	Control	1% LT	2% ST
(+)-catechin	0.89 ± 0.13 ^a^	1.22 ± 0.13 ^a^	7.95 ± 1.79 ^b^
(-)-epicatechin	0.20 ± 0.12 ^a^	0.61 ± 0.22 ^a^	3.14 ± 0.83 ^b^
(-)-epicatechin-3-O-methylether	1.85 ± 0.28 ^a^	4.21 ± 1.30 ^a^	15.59 ± 2.44 ^b^
(-)-epicatechin-4-O-methylether	1.63 ± 0.98 ^a^	1.28 ± 0.31 ^a^	2.36 ± 0.56 ^a^
m-coumaric acid	17.03 ± 1.96 ^a^	20.25 ± 6.46 ^a^	60.00 ± 17.47 ^b^
p-coumaric acid	5.76 ± 1.13 ^a^	6.03 ± 1.03 ^a^	12.96 ± 1.39 ^b^
2-hydroxybenzoic acid	22.57 ± 3.36 ^a^	20.37 ± 2.61 ^a^	23.62 ± 4.03 ^a^
3-hydroxybenzoic acid	43.60 ± 9.96 ^a^	84.05 ± 17.62 ^a^	1311.00 ± 363.10 ^b^
2.4-dihydroxybenzoic acid	4.80 ± 1.48 ^a^	6.37 ± 1.11 ^a^	3.98 ± 1.01 ^a^
4-hydroxybenzoic acid	17.81 ± 6.16 ^a^	9.44 ± 1.81 ^a^	11.35 ± 3.18 ^a^
Protocatechuic acid	8.07 ± 1.37 ^a^	5.88 ± 0.50 ^a^	10.00 ± 1.94 ^a^
Syringic acid	6.15 ± 0.74 ^a^	6.14 ± 1.53 ^a^	7.54 ± 1.56 ^a^
3-hydroxyphenylacetic acid	112.00 ± 4.33 ^a^	150.20 ± 20.72 ^a^	234.80 ± 14.80 ^b^

a,b in superscripts indicate significant differences between group (*p* < 0.05).

## Supplementary Materials

Supplementary materials can be found at https://www.mdpi.com/2076-2607/8/8/1141/s1. Supplemental Data S1: A scheme of the protocol for experimental trials. From the first day to the 4th week, all chicks received an ad libitum diet (free access to food). At 4th week, two groups of hens were separated in function of 2 types of diets: control diet and the same diet supplemented with GSE at 1% of total diet. At week 38 of age, a third group was created from the control group and received the diet supplemented with 2% GSE for two weeks. Supplemental Data S2: Oligonucleotide primer sequences.
