# Supplementary material for: Microbiota Changes Due to Grape Seed Extract Diet Improved Intestinal Homeostasis and Decreased Fatness in Parental Broiler Hens
Source: Microorganisms. 2020 Jul 28;8(8):1141. doi: 10.3390/microorganisms8081141 (PMC7465624; doi:10.3390/microorganisms8081141)
Supplement: Supplementary file 1 [file microorganisms-08-01141-s001.pdf]

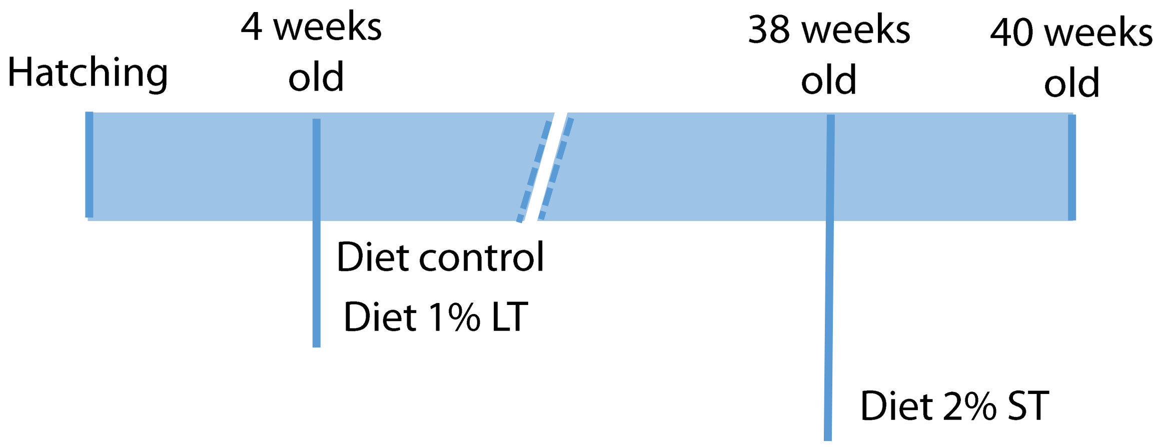

Supplemental Data 1. A scheme of the protocol for experimental trials.

From the first day to the 4th week, all chicks received an ad libitum diet (free access to food). At 4th week, two groups of hens were separated in function of 2 types of diets: control diet and the same diet supplemented with GSE at 1% of total diet. At week 38 of age, a third group was created from the control group and received the diet supplemented with 2% GSE for two weeks.

Gene	Sens	Reverse
ACTB	ACGGAACCACAGTTTATCATC	GTCCCAGTCTTCAACTATACC
EEF1A1	AGCAGACTTTGTGACCTTGCC	TCACATGAGACAGACGGTTGC
GAPDH	ACGGATTTGGTCGTATTGGG	TGATTTTGGAGGATCTCGC
IL22	TGTTGTTGCTGTTTCCCTCTTC	CACCCCTGTCCCTTTTGGA
mucin 2 (MUC-2)	GCTGATTGTCACTCACGCCTT	ATCTGCCTGAATCACAGGTGC
NOS2 (iNOS)	CCACCAGGAGATGTTGAACTATG TC	CCAGATGTGTGTTTTCCATGCA
IL1B	AGGCTCAACATTGCGCTGTA	CTTGTAGCCCTTGATGCCCA
TGFB1	CGCTCAGAACGACGTCAA	GTCGTCCACACCAACGAG
IgA	GTCACCGTCACCTGGACTACA	ACCGATGGTCTCCTTCACATC

Supplemental Data 2. Oligonucleotide primer sequences.
